# Burden of hypertension and associated risks for cardiovascular mortality in Cuba: a prospective cohort study

**DOI:** 10.1016/S2468-2667(18)30210-X

**Published:** 2019-01-23

**Authors:** Nurys Armas Rojas, Emily Dobell, Ben Lacey, Patricia Varona-Pérez, Julie Ann Burrett, Elba Lorenzo-Vázquez, Marcy Calderón Martínez, Paul Sherliker, Sonia Bess Constantén, José Manuel Morales Rigau, Osvaldo Jesús Hernández López, Miguel Ángel Martínez Morales, Ismell Alonso Alomá, Fernando Achiong Estupiñan, Mayda Díaz González, Noel Rosquete Muñoz, Marelis Cendra Asencio, Richard Peto, Jonathan Emberson, Alfredo Dueñas Herrera, Sarah Lewington

**Affiliations:** a National Institute of Cardiology and Cardiovascular Surgery, Havana, Cuba; b Clinical Trial Service Unit and Epidemiological Studies Unit (CTSU), Nuffield Department of Population Health, University of Oxford, Oxford, UK; c MRC Population Health Research Unit, University of Oxford, Oxford, UK; d Institute of Hygiene, Epidemiology and Microbiology, Ministry of Public Health, Havana, Cuba; e Cuban Commission against Smoking, Ministry of Public Health, Havana, Cuba; f Directorate of Medical Records and Health Statistics, Ministry of Public Health, Havana, Cuba; g Provincial Centre of Hygiene, Epidemiology and Microbiology, Matanzas, Cuba; h Municipal Centre of Hygiene, Epidemiology and Microbiology, Jagüey Grande, Matanzas, Cuba; i Municipal Centre of Hygiene, Epidemiology and Microbiology, Colón, Matanzas, Cuba; j Municipal Centre of Hygiene, Epidemiology and Microbiology, Camagüey, Cuba

## Abstract

**Background:**

In Cuba, hypertension control in primary care has been prioritised as a cost-effective means of addressing premature death from cardiovascular disease. However, there is little evidence from large-scale studies on the prevalence and management of hypertension in Cuba, and no direct evidence of the expected benefit of such efforts on cardiovascular mortality.

**Methods:**

In a prospective cohort study, adults in the general population identified via local family medical practices were interviewed between Jan 1, 1996, and Nov 24, 2002, in five areas of Cuba, and a subset of participants were resurveyed between July 14, 2006, and Oct 19, 2008, in one area. During household visits, blood pressure was measured and information obtained on diagnosis and treatment of hypertension. We calculated the prevalence of hypertension (systolic blood pressure ≥140 mm Hg or diastolic blood pressure ≥90 mm Hg, or receiving treatment for hypertension) and the proportion of people with hypertension in whom it was diagnosed, treated, and controlled (systolic blood pressure <140 mm Hg, diastolic blood pressure <90 mm Hg). Deaths were identified through linkage by national identification numbers to the Cuban Public Health Ministry records, to Dec 31, 2016. We used Cox regression analysis to compare cardiovascular mortality between participants with versus without uncontrolled hypertension. Rate ratios (RRs) were used to estimate the fraction of cardiovascular deaths attributable to hypertension.

**Findings:**

146 556 participants were interviewed in the baseline survey in 1996–2002 and 24 345 were interviewed in the resurvey in 2006–08. After exclusion for incomplete data and age outside the range of interest, 136 111 respondents aged 35–79 years (mean age 54 [SD 12] years; 75 947 [56%] women, 60 164 [44%] men) were eligible for inclusion in the analyses. 34% of participants had hypertension. Among these, 67% had a diagnosis of hypertension. 76% of participants with diagnosed hypertension were receiving treatment and blood pressure was controlled in 36% of those people. During 1·7 million person-years of follow-up there were 5707 cardiovascular deaths. In the age groups 35–59, 60–69, and 70–79 years, uncontrolled hypertension at baseline was associated with RRs of 2·15 (95% CI 1·88–2·46), 1·86 (1·69–2·05), and 1·41 (1·32–1·52), respectively, and accounted for around 20% of premature cardiovascular deaths.

**Interpretation:**

In this Cuban population, a third of people had hypertension. Although levels of hypertension diagnosis and treatment were commensurate with those in some high-income countries, the proportion of participants whose blood pressure was controlled was low. As well as reducing hypertension prevalence, improvement in blood pressure control among people with diagnosed hypertension is required to prevent premature cardiovascular deaths in Cuba.

**Funding:**

Medical Research Council, British Heart Foundation, Cancer Research UK.

## Introduction

Cuba is a middle-income country with universal health coverage.^1^ The health system focuses particularly on primary care and preventive medicine. This approach has delivered substantial reductions in infant and child mortality over the past few decades,^2^ but premature mortality in middle age (35–69 years) remains high. At 2015 mortality rates, 25% of men and 17% of women would die in middle age, mostly from non-communicable diseases, including about a third from cardiovascular disease.^3^

In 2013, WHO set global targets for the control of non-communicable diseases, including a 25% relative reduction in the prevalence of elevated blood pressure by 2025.^4^ Meta-analyses of prospective studies have shown that moderate differences in blood pressure have important implications for cardiovascular risk.^5^ Trials of blood-pressure-lowering medication have confirmed that much of the excess risk can be reversed within a few years of starting treatment.^6^, ^7^

In Cuba, hypertension control in primary care has been prioritised as a cost-effective means of addressing premature cardiovascular mortality.^8^ However, there are few large-scale studies on the prevalence and management of hypertension in Cuba, and there is no direct evidence from large prospective studies showing the expected benefit on cardiovascular mortality of efforts to improve hypertension control.

**Research in context**

**Evidence before this study**

We did a literature search to identify cross-sectional or prospective studies done in Cuba reporting on the prevalence of hypertension or its associated risks for cardiovascular disease. We searched PubMed for articles published between Jan 1, 1960, and Feb 2, 2018, in any language, using the search string “Cuba (OR Cuban) AND hypertension (OR blood pressure) AND cross-sectional study (OR prospective study OR cohort study)”. We also searched the reference lists of retrieved articles to identify further relevant publications. We identified several small studies (<5000 adults) and two larger studies (a national survey in 2001 of 23 000 adults living in urban areas and a regional survey of 55 000 adults in 2004–06). Neither of the larger studies reported the proportions of people who had diagnosed hypertension, were receiving treatment, or in whom blood pressure was controlled or assessed the excess cardiovascular risks of uncontrolled hypertension.

**Added value of this study**

This study is to our knowledge the largest prospective analysis of hypertension-related mortality done in Cuba, and one of the largest in Latin America. The prevalence, diagnosis, and management of hypertension are assessed by age, sex, education, area, and previous diagnosis of cardiovascular disease. We have also assessed the age-specific and sex-specific effects of uncontrolled hypertension on cardiovascular mortality. At ages 35–79 years, about a third of participants had hypertension. The proportions of people in whom hypertension was diagnosed and being treated were commensurate with those in some high-income countries, but control of blood pressure was low. Overall, about 80% of all people with hypertension had uncontrolled blood pressure, which was associated with roughly a doubling of risk of premature cardiovascular death.

**Implications of all the available evidence**

Addressing the burden of hypertension in Cuba will require not only improved detection of hypertension in primary care but also greater control of blood pressure among people with hypertension. Several initiatives using community-wide awareness campaigns and simplified hypertension treatment regimens are ongoing in Cuba, the findings of which will be relevant nationally and to other low-income and middle-income countries. Cuba's health system, which focuses on primary care and preventative medicine, has delivered substantial reductions in infant and child mortality over the past few decades, but death from the chronic diseases of middle age remains high. Hypertension control in primary care has been prioritised as a cost-effective means of addressing premature death in Cuba, and this study helps to inform the delivery of such programmes and estimate their potential impact on cardiovascular mortality.

We report a large prospective study of the burden of hypertension and its associated risks of premature cardiovascular mortality in Cuba. In reporting, we had several aims: to describe the prevalence, diagnosis, and management of hypertension by age, sex, education, area, and previous diagnosis of cardiovascular disease; assess the age-specific and sex-specific effects of uncontrolled hypertension on cardiovascular mortality; and estimate the proportion of cardiovascular deaths attributable to hypertension in Cuba.

## Methods

### Study design and participants

This was a prospective cohort study that, from Jan 1, 1996, to Nov 24, 2002, recruited members of the general population in five areas of Cuba, including the capital city (Havana) and four geographically dispersed provinces (Pinar del Río, Matanzas, La Habana, and Camagüey). The populations of these areas collectively accounted for just under half of Cuba's population in 2000. Within each area, family medical practices were randomly selected with use of a computer-generated random allocation sequence, and local health-care staff (mostly physicians) were asked to recruit all residents aged 30 years or older within their catchment area. On average, each practice provided care for around 150 families.^2^

Ethics approval was provided by the National Institute of Cardiology and Cardiac Surgery in Havana. All participants provided written informed consent.

### Study procedures

Trained health-care staff from the family practices made household visits and invited eligible household members to participate in the study. They recorded age, sex, education, occupation, information on health-related behaviours (including smoking and alcohol intake), and medical history. Blood pressure was measured twice while the participant was seated, using standard techniques and a calibrated manual sphygmomanometer. The mean of the two measurements was used in our analyses. Height and weight were measured separately at the medical practice because the mechanical scales commonly used at the time of survey in Cuba were not easily portable. The original data collection form was in Spanish, but an English translation is available in the appendix.

In 2006–08, participants in two municipalities (Colón and Jagüey Grande) in the province of Matanzas who had participated in the first survey were invited to be resurveyed. The same procedures were used as for the baseline survey.

Deaths were identified through linkage by national identification numbers to the Cuban Public Health Ministry records until Dec 31, 2016. We were able to capture deaths for participants who moved out of the study areas to other parts of Cuba, but not for those who emigrated. The underlying causes of death were coded according to the International Classification of Diseases ninth and tenth editions (ICD-9 and ICD-10). We used ICD-9 codes for deaths between 1996 and 2000, and ICD-10 codes for deaths from 2001 onwards. Cardiovascular deaths were defined as deaths from myocardial infarction, stroke, or other vascular disease (ICD-9 codes 390–459, and 798, and ICD-10 codes I00–I99, and R96).

### Statistical analysis

The analyses excluded participants with any missing data on the demographic characteristics of age, sex, education, and area, missing or implausible blood pressure values, and age outside the range of interest (35–79 years). Mean systolic blood pressure (SBP), mean diastolic blood pressure (DBP), and prevalence of hypertension were calculated for men and women separately in the age groups 35–39, 40–49, 50–59, 60–69, and 70–79 years. We classified participants as having hypertension if they had SBP 140 mm Hg or greater or DBP 90 mm Hg or greater on measurement at baseline or if they reported that they had a diagnosis of hypertension and were receiving blood-pressure-lowering medication (irrespective of measured blood pressure at baseline).^9^

To assess the management of hypertension, we calculated the proportion of people at baseline who had hypertension diagnosed by a doctor, the proportion of those diagnosed who were being treated with blood-pressure-lowering medication, and the proportion of those receiving treatment who had controlled blood pressure (SBP <140 mm Hg and DBP <90 mm Hg). These proportions were estimated overall and by age, sex, area, education, ethnicity, history of cardiovascular disease, and season of baseline survey (because seasonal variation in blood pressure has been found in some populations^10^). Analyses were standardised, where appropriate, for age, sex, and area.

Cox regression was used to calculate rate ratios (RRs) and 95% CIs for cardiovascular death among participants with and without uncontrolled hypertension. To limit reverse causality, these analyses excluded people with previously diagnosed cardiovascular disease. RRs were estimated by sex and age at risk (age groups 35–59, 60–69, and 70–79 years) and adjusted for age at risk in 5-year age groups, area, and level of education. The regression analysis used time in study as the underlying time variable and adjusted for current age by fitting a term that allows the RR in each decade of age to be estimated as the geometric mean of the RRs in the first and second half of that decade.

Age-specific and sex-specific population-attributable fractions were calculated with the equation

$${\text{P}}_{\text{e}}(\text{RR}-1)/\text{RR}$$

where P_e_ is the proportion of cardiovascular deaths occurring in participants with uncontrolled hypertension and RR is the adjusted cardiovascular death RR in participants with versus those without uncontrolled hypertension.^11^ Estimates of the number of deaths attributable to uncontrolled hypertension in Cuba were made by applying the population-attributable fractions to the age-specific and sex-specific number of deaths from cardiovascular disease in 2015, using data from the Global Burden of Disease study.^3^ All analyses were done with SAS version 9.3, and figures were plotted with R version 2.14.1.

### Role of the funding source

The funders of the study had no role in the study design, data collection, data analysis, data interpretation, or writing of the report. The corresponding author had full access to all the data and had final responsibility for the decision to submit for publication.

## Results

Of 215 practices approached, none refused to participate. Between Jan 1, 1996, and Nov 24, 2002, of 198 049 residents in the catchment areas, 146 556 (74%) men and women were interviewed in the first survey. We excluded 164 participants with missing demographic information, 68 with missing or implausible blood pressure values, and 10 111 outside the age range of interest, leaving 136 111 participants eligible for inclusion in the analyses (table 1). The mean age of participants was 54 (SD 12) years, just over half were women, and most were white. Almost all participants (127 355 [94%]) had received some formal education, and two-thirds (89 460 [66%]) were educated to secondary school level or higher. Most participants were recruited from Matanzas in Central Cuba and Camagüey in eastern Cuba. 35 703 (59%) men smoked (cigarettes or cigars) and 17 311 (29%) drank alcohol at least weekly. By contrast, 25 025 (33%) women smoked and only 3434 (5%) drank at least weekly. Overall, mean SBP, DBP, and body-mass index were similar in men and women. By Dec 31, 2016, 26 465 (19%) participants had died and 204 (<1%) were lost to follow-up through emigration or uncertainty about the national identification number provided at baseline.

**Table 1:** Baseline characteristics

	**Men (n=60 164)**	**Women (n=75 947)**	**All (n=136 111)**
**Age at entry (years)**			
35–39	9291 (15%)	12 301 (16%)	21 592 (16%)
40–49	16 774 (28%)	21 651 (29%)	38 425 (28%)
50–59	15 347 (26%)	19 014 (25%)	34 361 (25%)
60–69	11 192 (19%)	13 920 (18%)	25 112 (18%)
70–79	7560 (13%)	9061 (12%)	16 621 (12%)
Mean (SD)	54 (12)	53 (12)	54 (12)
**Blood pressure (mm Hg)**			
Systolic	126 (14)	124 (16)	125 (15)
Diastolic	81 (9)	80 (10)	80 (10)
**Area of Cuba**			
Matanzas	26 778 (45%)	32 877 (43%)	59 655 (44%)
Camagüey	24 160 (40%)	30 619 (40%)	54 779 (40%)
Pinar del Río	4873 (8%)	5903 (8%)	10 776 (8%)
Ciudad de La Habana	3088 (5%)	4946 (7%)	8034 (6%)
La Habana	1265 (2%)	1602 (2%)	2867 (2%)
**Educational level**			
Less than primary	3240 (5%)	5516 (7%)	8756 (6%)
Primary	14 758 (25%)	23 137 (30%)	37 895 (28%)
Lower secondary	16 142 (27%)	21 106 (28%)	37 248 (27%)
High school/technical college	20 068 (33%)	19 637 (26%)	39 705 (29%)
University	5956 (10%)	6551 (9%)	12 507 (9%)
**Ethnicity**			
White	46 605 (77%)	58 307 (77%)	104 912 (77%)
Black	8763 (15%)	10 791 (14%)	19 554 (14%)
Mixed	4596 (8%)	6597 (9%)	11 193 (8%)
Other	200 (<1%)	252 (<1%)	452 (<1%)
**Previous cardiovascular disease**			
No	57 782 (96%)	73 664 (97%)	131 446 (97%)
Yes	2382 (4%)	2283 (3%)	4665 (3%)
**Season of baseline survey**			
Spring	12 649 (21%)	16 242 (21%)	28 891 (21%)
Summer	19 612 (33%)	23 812 (31%)	43 424 (32%)
Autumn	12 151 (20%)	16 055 (21%)	28 206 (21%)
Winter	15 752 (26%)	19 838 (26%)	35 590 (26%)

Data are n (%) or mean (SD). Data exclude participants with missing demographic information, missing or implausible blood pressure values, and age outside the range of interest (35–79 years).

**Figure 1:**
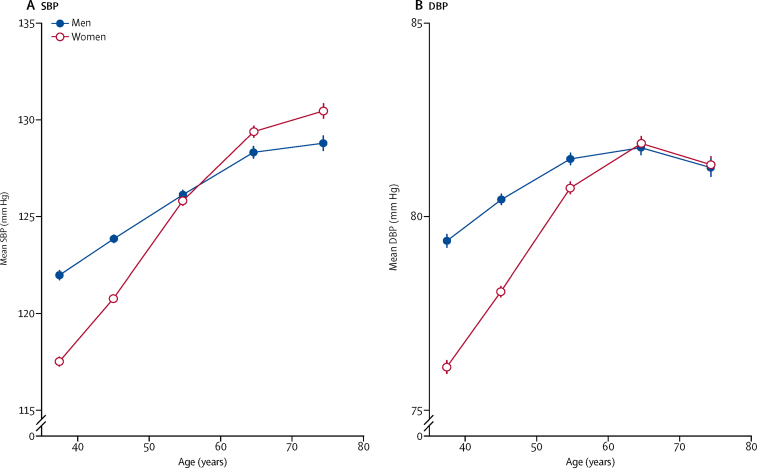
Mean blood pressure, by age and sex (A) SBP. (B) DBP. Data are mean (95% CI). Means are standardised for area. Analyses in 136 111 participants. DBP=diastolic blood pressure. SBP=systolic blood pressure.

We found a positive and roughly linear association between age and mean SBP at baseline in both sexes (figure 1). Mean SBP was higher in men than women at younger ages but increased more steeply with age in women than men, and was higher in women from around age 65 years and older. By contrast, the association of mean DBP with age was non-linear. As with SBP, mean DBP was higher in men than women at younger ages and increased more steeply with age in women, but from age 65 years there was little association with age and no difference between the sexes.

**Figure 2:**
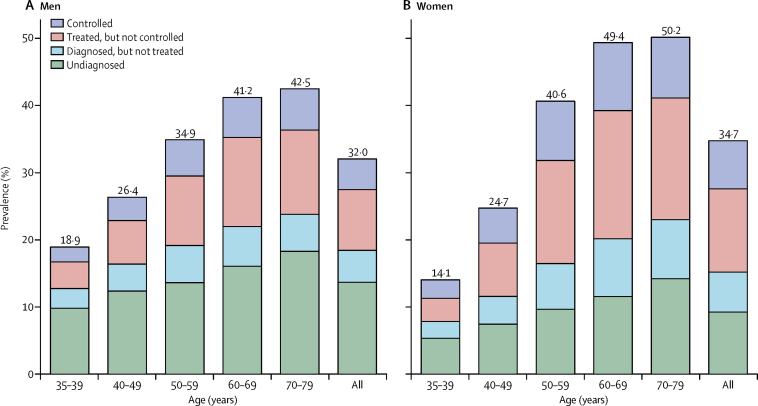
Prevalence of hypertension, by age and sex (A) Men. (B) Women. Controlled hypertension at baseline is defined as systolic blood pressure less than 140 mm Hg and diastolic blood pressure less than 90 mm Hg. Prevalence is standardised for area and, where appropriate, age The analysis included 136 111 participants. All=participants aged 35–79 years.

Overall, about a third of adults had hypertension (32% of men and 35% of women; table 2, figure 2). 6% had isolated systolic hypertension, 8% isolated diastolic hypertension, and 14% both systolic and diastolic hypertension, and 6% had controlled hypertension (appendix). 20% of all participants had blood pressure consistent with stage 1 hypertension (SBP 140–159 mm Hg, DBP 90–99 mm Hg, or both^12^) and 7% had higher blood pressure values (appendix). The prevalence of hypertension increased substantially with age in both sexes and was higher in men than women at younger ages (19% in men and 14% in women aged 35–39 years) but higher in women than men at older ages (50% in women and 43% in men at age 70–79 years; appendix).

**Table 2:** Prevalence, diagnosis, treatment, and control of hypertension, by age and sex*

	**Number of participants**	**Prevalence of hypertension**	**Proportion with diagnosed hypertension**	**Proportion of diagnosed patients treated**	**Controlled hypertension**	
					Among treated	Among all hypertensives
**Men**						
35–39 years	9291	18·9%	48·2%	67·7%	36·0%	11·7%
40–49 years	16 774	26·4%	53·0%	71·2%	34·8%	13·1%
50–59 years	15 347	34·9%	60·9%	74·0%	34·2%	15·4%
60–69 years	11 192	41·2%	61·0%	76·4%	31·0%	14·5%
70–79 years	7560	42·5%	57·0%	77·1%	33·0%	14·5%
All	60 164	32·0%	57·3%	74·1%	33·4%	14·2%
**Women**						
35–39 years	12 301	14·1%	62·0%	71·4%	44·4%	19·6%
40–49 years	21 651	24·7%	69·9%	76·1%	39·6%	21·0%
50–59 years	19 014	40·6%	76·2%	78·1%	36·6%	21·8%
60–69 years	13 920	49·4%	76·6%	77·2%	34·7%	20·5%
70–79 years	9061	50·2%	71·7%	75·5%	33·4%	18·1%
All	75 947	34·7%	73·4%	76·7%	36·5%	20·6%
**Men and women**						
35–39 years	21 592	16·2%	54·9%	69·7%	40·7%	15·5%
40–49 years	38 425	25·5%	62·1%	74·2%	37·8%	17·4%
50–59 years	34 361	38·1%	70·0%	76·7%	35·8%	19·2%
60–69 years	25 112	45·8%	70·4%	76·9%	33·5%	18·1%
70–79 years	16 621	46·8%	65·8%	76·1%	33·2%	16·6%
Overall	136 111	33·5%	66·5%	75·8%	35·5%	17·9%

* Proportions are standardised for area and, where appropriate, age and sex.

Among participants with hypertension at baseline, 67% had a diagnosis of hypertension and among these 76% were receiving treatment. In 36% of participants receiving treatment, blood pressure was controlled (table 2). Overall, 18% of participants with hypertension (14% of men and 21% of women) had controlled blood pressure (table 2). The proportion of participants with diagnosed hypertension increased with increasing age, but at all ages was higher in women than men (table 2). The proportion of participants with diagnosed hypertension who were treated also increased with age but did not differ substantially between the sexes. By contrast, control of hypertension among people receiving treatment declined with age, but was low even at younger ages in both men and women (table 2).

69% of participants treated for hypertension were taking one blood-pressure-lowering medication, 28% were taking two medications, and 3% were taking three medications (appendix). 61% of treated participants were taking diuretics (27% in combination with other antihypertensive drugs and 34% alone) and only 5% of all patients being treated for hypertension were taking angiotensin-converting-enzyme inhibitors (appendix).

The prevalence of hypertension did not differ greatly by survey area, educational level, or season of survey (table 3), but was somewhat higher among black participants and those with mixed ethnicity than white participants, and was substantially higher among those with previous cardiovascular disease than those without. The proportion of all participants with hypertension in whom blood pressure was controlled varied by area, from 14% in Matanzas to 27% in Pinar del Río, and was slightly greater among participants with higher levels of education, those of white ethnicity, and those with previous cardiovascular disease (table 3). However, even among participants with previous cardiovascular disease, blood pressure was controlled in only 26%.

**Table 3:** Prevalence, diagnosis, treatment, and control of hypertension* by selected characteristics†

		**Number of participants**	**Prevalence of hypertension**	**Proportion with diagnosed hypertension**	**Proportion of diagnosed patients treated**	**Controlled hypertension**	
						Among treated	Among all hypertensives
Area							
	Matanzas	59 655	34·5%	62·3%	69·8%	31·6%	13·8%
	Camagüey	54 779	32·5%	67·9%	77·5%	37·3%	19·6%
	Pinar del Río	10 776	32·2%	77·8%	89·0%	38·8%	26·7%
	Ciudad de la Habana	8034	35·7%	75·3%	83·6%	39·8%	25·0%
	La Habana	2867	31·3%	63·1%	76·4%	36·9%	17·9%
Educational level							
	Less than primary	8756	33·5%	61·8%	74·9%	34·8%	16·5%
	Primary	37 895	34·3%	64·9%	75·0%	34·9%	17·0%
	Lower secondary	37 248	33·0%	66·5%	75·8%	35·5%	18·0%
	High school/technical college	39 705	32·8%	67·6%	76·2%	38·2%	19·6%
	University	12 507	33·2%	70·0%	76·6%	39·8%	21·3%
Ethnicity							
	White	104 912	31·7%	65·6%	75·7%	37·3%	18·6%
	Black	19 554	41·8%	69·4%	75·9%	29·2%	15·4%
	Mixed	11 193	36·5%	67·4%	75·5%	32·2%	16·5%
	Other	452	32·8%	65·9%	65·6%	27·7%	12·8%
Previous cardiovascular disease							
	No	131 446	32·8%	65·5%	75·1%	35·2%	17·4%
	Yes	4665	58·1%	81·9%	83·7%	37·7%	26·0%
Season of baseline survey							
	Spring	28 891	35·1%	63·0%	77·2%	32·9%	16·1%
	Summer	43 424	32·1%	66·7%	74·9%	35·8%	18·0%
	Autumn	28 206	33·1%	69·7%	80·0%	38·7%	21·5%
	Winter	35 590	35·7%	66·4%	74·8%	33·6%	16·7%
Overall		136 111	33·5%	66·5%	75·8%	35·5%	17·9%

* Hypertension is categorised as undiagnosed, diagnosed but not treated, treated but not controlled, or controlled (ie, blood pressure at baseline <140 mm Hg systolic and < 90 mm Hg diastolic).

† Proportions are standardised for age, sex, and, where appropriate, area.

The resurvey was done between July 14, 2006, and Oct 19, 2008. Of 27 983 participants invited to take part, 24 345 (87%) were interviewed. After exclusions, 23 114 were included in the analysis (appendix). The characteristics of people resurveyed were similar to those of the cohort as a whole (appendix). The prevalence of hypertension among participants aged 40–79 years (age-standardised to the baseline findings) was unchanged from baseline (39% at resurvey *vs* 38% at baseline), as were the proportions of those with hypertension that were diagnosed and being treated (appendix). By contrast, the proportion of participants with treated hypertension who had controlled blood pressure had increased from 36% in the first survey to 59% in the resurvey. This difference was seen despite only a slight increase in the proportion of participants receiving more than one blood-pressure-lowering medication (from 31% at baseline to 36%, appendix).

**Figure 3:**
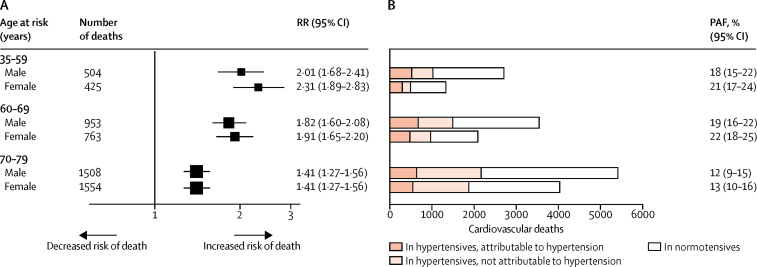
RRs for deaths in people with uncontrolled hypertension and cardiovascular mortality in Cuba for 2015 (A) RRs for death are calculated for participants with versus those without uncontrolled hypertension, and are adjusted for area, level of education, and age within each age group. For each RR, the area of the square is inversely proportional to the variance of the log RR. (B) Cardiovascular mortality attributable to uncontrolled hypertension is calculated by applying PAFs to the estimated age-specific and sex-specific number of cardiovascular deaths in Cuba for 2015. PAF=population-attributable fraction. RR=rate ratio.

During 1·7 million person-years of follow-up (mean 17 [SD 4] years per person), 5707 cardiovascular deaths occurred in participants aged 35–79 years (figure 3). Uncontrolled hypertension at baseline was associated with RRs of 2·15 (95% CI 1·88–2·46), 1·86 (1·69–2·05) and 1·41 (1·32–1·52) at ages 35–59, 60–69, and 70–79 years, respectively. RRs were similar in men and women and were not changed substantially by further adjustment for education, smoking, alcohol intake, or body-mass index (appendix). The excess cardiovascular mortality associated with uncontrolled hypertension at baseline accounted for 20% (95% CI 18–22) of all cardiovascular deaths in the age group 35–69 years, and 13% (10–15) in the age group 70–79 years (figure 3). These population-attributable proportions would be equivalent to around 2000 excess cardiovascular deaths due to uncontrolled hypertension among people aged 35–69 years and around 1200 among those aged 70–79 years in Cuba in 2015.

## Discussion

In the Cuban population we assessed, a third of participants had hypertension. Two-thirds of those participants had a previous diagnosis, among whom three-quarters were receiving treatment. However, only about a third of treated patients had controlled blood pressure. The prevalence of hypertension was strongly related to age and was higher in men than women at younger ages but higher in women than men at older ages. Blood pressure was not well controlled in any subgroup, even in participants with previous cardiovascular disease. Uncontrolled hypertension was estimated to account for 20% of premature cardiovascular deaths in 2015 in this cohort.

Although the study was not designed to be nationally representative, the sociodemographic characteristics of participants in the baseline survey (1996–2002) and the estimated prevalence of hypertension are consistent with a national survey of risk factors for chronic disease that was done in Cuba in 2001,^13^ which supports the generalisability of our findings. The national survey involved 23 000 participants (mean age 44 years) and found that 34% of adults had hypertension.^13^, ^14^ Another national survey done among adults in 2010 (mean age 46 years), found that prevalence of hypertension was largely unchanged (31%).^13^, ^14^ These data concur with our finding of similar hypertension prevalence at the time of the resurvey in 2006–08 (34%). The proportions of people with hypertension that was diagnosed, treated, and controlled were not reported in the national studies, and such results are only available from a few cross-sectional surveys in local areas,^15^, ^16^, ^17^ which cannot be generalised reliably to Cuba as a whole.

The age-specific prevalence values we report for hypertension are somewhat lower than those reported in surveys in many high-income countries, including those in western Europe and North America, even when more inclusive definitions of hypertension are used (appendix). Our values are, however, higher than in some other populations, such those in parts of east Asia.^18^ The proportion of participants in this study with hypertension that had been diagnosed and treated is broadly consistent with some high-income countries, despite the more limited resources of the Cuban health system.^19^ However, our finding that only 36% of treated participants with hypertension had controlled blood pressure was much lower than has generally been reported in high-income countries, such as 50% in the USA and 66% in Canada.^19^

Meta-analyses of randomised clinical trials of blood-pressure-lowering medication have shown that vascular risk is lowered by reducing SBP to 140 mm Hg and DBP to 90 mm Hg.^6^, ^20^ Some trials, however, that have involved selected populations, have found benefits with further reductions.^21^, ^22^, ^23^ Achieving controlled blood pressure in people with hypertension often requires several blood-pressure-lowering medications, but in our study most participants with treated hypertension at baseline and at the time of the resurvey were taking only one blood-pressure-lowering medication.

The undertreatment of hypertension at baseline and resurvey was unexpected, given that Cuba's health system focuses on preventive medicine in primary care. This finding might in part reflect the availability of blood pressure-lowering medications. Shortages of common medications have occurred several times over the past few decades in Cuba, even in 2017,^24^ mainly during periods of economic hardship. Indeed, the baseline survey was done immediately following a time of economic crisis in Cuba that began in 1991.^1^

We found that uncontrolled hypertension was associated with roughly a doubling of the risk of premature death from cardiovascular disease. Meta-analyses of prospective studies, however, have shown direct and continuous associations between blood pressure and cardiovascular mortality, with no evidence of a threshold down to at least SBP 115 mm Hg.^5^ As such, the excess risk of cardiovascular death reflects the mean difference in the long-term average blood pressure between people with uncontrolled hypertension and those with controlled blood pressure (roughly 20 mm Hg systolic in the present study). Equivalent reductions in blood pressure within the hypertensive range would be expected to have the same effect on cardiovascular risk.

The strengths of the present study include the very large sample size, the diverse areas surveyed, the range of population subgroups assessed, the stable population with low loss to follow-up, and the reliable linkage to cause-specific mortality. It is a limitation of the study, however, that our analyses could only describe associations with mortality because data were not available for non-fatal myocardial infarction and stroke events. Furthermore, hypertension was identified by measurement of blood pressure on one occasion at baseline. This is the standard approach in epidemiological surveys but differs from that for the diagnosis of hypertension in clinical practice, which is usually based on blood pressure recorded on at least two separate occasions. Additionally, any real improvements in blood pressure control during follow-up are likely to have attenuated the associations between uncontrolled hypertension at baseline and cardiovascular disease. Finally, the resurvey was done in only two municipalities. The initial plan had included more areas, but the number had to be reduced due to resource limitations.

Addressing the burden of hypertension in Cuba will require not only improved detection of hypertension in primary care (as in high-income countries), but also greater control of blood pressure among people known to have hypertension. The people with most to gain from lowering of blood pressure would be those at greatest absolute risk of hypertension (ie, those with previous cardiovascular disease or those with diabetes, who would also benefit from other treatments to address cardiovascular risk, such as statins, irrespective of blood pressure). The findings from our resurvey indicate that blood pressure control might have improved in some regions of Cuba, and an ongoing national survey will provide evidence on whether effects are similar at the national level, including for secondary prevention.

Several initiatives using community-wide awareness campaigns and simplified hypertension treatment regimens are ongoing in Cuba,^25^ and our findings should provide useful insights into addressing uncontrolled hypertension in primary care. Further research is also required to develop a cardiovascular risk score that is validated in the general population in Cuba to identify those at high absolute risk of cardiovascular events. Ideally, such efforts to improve the management of hypertension in primary care should be accompanied by public health programmes at the local level, national level, or both, to address the major determinants of hypertension, including harmful alcohol intake, excess adiposity, and high salt intake. The latter factor has been promoted by the Pan American Health Organization as an important public health measure to address raised blood pressure in the Americas.^26^

In this Cuban cohort study, a third of participants at baseline and resurvey had hypertension. Although the proportions of participants with diagnosed and treated hypertension were commensurate with those in some high-income countries, overall control of blood pressure was low. In addition to public health measures to reduce the prevalence of hypertension, improvement of blood-pressure control among people with diagnosed hypertension is required to prevent premature cardiovascular deaths in Cuba.

**This online publication has been corrected. The corrected version first appeared at thelancet.com/public-health on February 6, 2019**
