# Correction to *Lancet Public Health* 2019; 4: e107–15

**DOI:** 10.1016/S2468-2667(19)30004-0

**Published:** 2019-02-06

*Armas Rojas N, Dobell E, Lacey B, et al. Burden of hypertension and associated risks for cardiovascular mortality in Cuba: a prospective cohort study.* Lancet Public Health *2019; **4:** e107–15*—The declaration of interests statement for JE should have been “JE received a grant from the Medical Research Council during the conduct of the study, and grants from the British Heart Foundation and Boehringer Ingelheim outside the study”. This correction has been made to the online version as of Feb 6, 2019.

