# Supplementary material for: Burden of hypertension and associated risks for cardiovascular mortality in Cuba: a prospective cohort study
Source: Lancet Public Health. 2019 Jan 23;4(2):e107–15. doi: 10.1016/S2468-2667(18)30210-X (PMC6365887; doi:10.1016/S2468-2667(18)30210-X)
Supplement: Supplementary appendix [file mmc1.pdf]

THE LANCET
Public Health

Supplementary appendix

This appendix formed part of the original submission and has been peer reviewed. We post it as supplied by the authors.

Supplement to: Rojas NA, Dobell E, Lacey B, et al. Burden of hypertension and associated risks for cardiovascular mortality in Cuba: a prospective cohort study. *Lancet Public Health* 2019; published online Jan 22. http://dx.doi.org/10.1016/S2468-2667(18)30210-X.

This page intentionally left blank

Burden of hypertension and associated risks for cardiovascular mortality in Cuba: a prospective study of 150,000 adults

Supplementary Appendix, Table of Contents

	Page
**Questionnaire** (Spanish)	3
**Questionnaire** (English translation)	4
**Table S1.** Prevalence of isolated systolic hypertension and isolated diastolic hypertension, by age and sex	5
**Table S2.** Prevalence of stage 1 hypertension and stage 2 hypertension, by age and sex	6
**Table S3.** Proportion of participants with treated hypertension taking a single or multiple blood pressure-lowering medications, by age and sex	7
**Table S4.** Proportion of participants with treated hypertension taking specific types of blood pressure-lowering medication, by age and sex	8
**Table S5.** Baseline characteristics of the 23 114 study participants that were resurveyed	9
**Table S6.** Prevalence, diagnosis, treatment and control of hypertension at baseline among resurveyed participants, by age and sex	10
**Table S7.** Prevalence, diagnosis, treatment and control of hypertension at among resurvey participants, by age and sex	11
**Table S8.** Number of hypertensive participants taking 1,2,3 or 4 antihypertensives at resurvey, by age and sex	12
**Table S9.** Baseline characteristics of the 127 253 study participants in the prospective analyses	13
**Figure S2.** Age- and sex-specific relevance of hypertension to cardiovascular mortality	14
**Table S10.** Prevalence and control of hypertension using four different definitions of hypertension	15

ESTUDIO CUBANO SOBRE TABACO Y SALUD

Provincia No. ☐ 1-9 Municipio No. ☐☐ 0-99 Area de Salud No. ☐ 0-9

№ 17524 A

Carnet de Identidad No. |_|_|_|_|_|_|–|_|_|_|_|_|
Fecha de nacimiento (a.m.d) + 5 números
(11 números: excluir si no tiene C.I. de 6 + 5 números)

Nombre de la persona y dirección: |_|_|_|_|_|_|_|_|_|_|_|_|_|_|_|_|_|_|_|_|_|_|_|_|_|
Calle No. Teléfono

Nombre del familiar y dirección: |_|_|_|_|_|_|_|_|_|_|_|_|_|_|_|_|_|_|_|_|_|_|_|_|_|
Calle No. Municipio Provincia Teléfono

LETRA DE MOLDE, POR FAVOR

Sexo (1 = masc, 2 = fem.) ☐ 1-2

Color de piel (1 = blanco, 2 = negro, 3 = mestizo, 4 = otra) ☐ 1-4

Estado social (1 = soltero/viudo, 2 = casado) ☐ 1-2

Educación completada (0 = ninguno*, 1 = primaria, 2 = secundaria, 3 = técnico medio, 4 = obrero calificado, 5 = pre-universitario, 6 = universitario) ☐ 0-6

Categoría ocupacional (0 = ninguno*, 1 = dirigente, 2 = profesional, 3 = técnico, 4 = de servicio, 5 = agrícola, 6 = industria, 7 = militar) ☐ 0-7

* Si es cero (Ej. ama de casa, jubilado o desempleado), diga si ha trabajado alguna vez. ☐ 0-7

Quisiera medir su presión arterial mientras esta sentado, hacerle unas pocas preguntas sobre tabaco y alcohol, y medir su PA de nuevo.

Presión arterial (una sola toma, sentado): |_|_|_| / |_|_|_| PAS / PAD

Ahora, TABACO: ¿Ha fumado alguna vez regularmente (Ej. en la mayoría de los días)?
SI NO, vaya directamente a las preguntas sobre ALCOHOL. ☐ S=Sí, N=No

¿A QUE EDAD APROXIMADAMENTE COMENZO USTED A FUMAR REGULARMENTE? ☐☐ años de edad

¿Desde entonces ha dejado de fumar alguna vez por un año o más? ☐ S=Sí, N=No

¿Aproximadamente cuántos cigarrillos por día se fumó usted el último mes? (0 = ninguno) ☐☐ diariamente

¿Aproximadamente cuántos tabacos por día se fumó usted el último mes? (0 = ninguno) ☐☐ diariamente

¿Si no fumó el último mes, a que edad dejó usted de fumar? (en blanco si no es aplicable) ☐☐ años de edad

Ahora me gustaría hacerle algunas preguntas sobre ALCOHOL:

¿Aproximadamente cuántos días a la semana toma usted ron u otra bebida fuerte? (0 = cero días o usualmente menos de una vez por semana) ☐ 0-7

¿El día que usted toma ron (u otra bebida fuerte) cuántas líneas se toma? ☐☐ líneas

¿Aproximadamente cuántos días a la semana toma usted cerveza? (0 = cero días o usualmente menos de una vez por semana) ☐ 0-7

¿El día que usted toma cerveza, cuántas botellas se toma (aprox.) ☐☐ botellas

Finalmente, algunos aspectos médicos: Quisiera preguntarle sobre algunas enfermedades. Lo que quiero conocer es si usted alguna vez ha sido tratado por alguna de estas enfermedades. Si no lo ha sido, conteste No (N).

Asma	☐ S/N	A.V.E. (definitivo/probable)	☐ S/N	Cirrosis	☐ S/N
Enfisema (E.P.O.C.)	☐ S/N	Infarto miocardio "	☐ S/N	Enf. renal crónica	☐ S/N
Diabetes	☐ S/N	Angina pectoris "	☐ S/N	Cáncer de piel	☐ S/N
Ulcera péptica	☐ S/N	Hipertensión arterial	☐ S/N	Otro cáncer*	☐ S/N

¿*Si Otro Cáncer = Sí, dónde le comenzó el cáncer? ☐ 0-9

(1 = boca o faringe, 2 = laringe, 3 = pulmón, 4 = esófago, 5 = estómago, 6 = páncreas, 7 = cuello útero, 8 = vejiga, 9 = mama, 0 = otro, más de un sitio, o no conocido)

¿Esta usted tomando alguna medicina regularmente desde hace más de un mes? Si es Sí: ¿Qué medicinas esta tomando? (Marque con X)

Digoxina	☐	Nitratos	☐	Hipoglic. oral	☐	Anticoncept.	☐	Ca. Antag.	☐
Beta B.	☐	Inh. ACE	☐	Insulina	☐	Antidepres.	☐	Broncodilat.	☐
Diurético	☐	ASA	☐	Esteroides	☐	Ansiolítico	☐	Antiácido/H2	☐

MUCHAS GRACIAS POR SUS RESPUESTAS: Ahora necesito tomarle de nuevo su presión arterial. Repita la toma de PA (una sola toma sentado): |_|_|_| / |_|_|_| PAS/PAD

¿Cuál es la última cifra PA que tiene en su historia clínica antes de hoy?

Fecha de la última PA (a.m.d) |_|_|_|_|_|_| & |_|_|_| / |_|_|_| PAS/PAD

¿Cuál es su *TALLA* aproximada? (medirlo o preguntarlo) |_|_|_| cm

¿Cuál es su *PESO* aproximado? (pesarlo o preguntarlo) |_|_|_| kg

Fecha de la entrevista |_|_|_|_|_|_| (a.m.d)

Por favor chequee TODA la encuesta ¡MUCHAS GRACIAS!

Calidad de la cooperación (1 = pobre, 2 = regular, 3 = buena) ☐ 1-3

Nombre y apellidos del entrevistador: |_|_|_|_|_|_|_|_|_|_|_|_|_|_|_|_|_|_|

¿Algún comentario? ....

PARA LA SIGUIENTE PERSONA, UTILICE EL REVERSO DE ESTA PAGINA →

CUBAN STUDY OF TOBACCO AND HEALTH: PROSPECTIVE

Province Nº ☐ 1-9 Municipality Nº ☐☐ 0-99 Health Area Nº ☐ 0-9 Identity Card Nº ☐☐☐☐☐☐-☐☐☐☐☐ Date of birth (y,m,d)

Name of interviewee: ____________________

Address: ____________________
Street Nº Municipality Province Telephone

Name of friend: ____________________

Address: ____________________
Street Nº Municipality Province Telephone

CAPITAL LETTERS, PLEASE!

Sex (1=masc, 2=fem) ☐ 1-2

Skin colour(1=white, 2=black, 3=mixed, 4=other) ☐ 1-4

Marital status (1=single/widowed, 2=married) ☐ 1-2

Education completed (0=none, 1=primary, 2=secondary, 3=technician, 4=skilled worker, 5=high school, 6=university) ☐ 0-6

Occupational category (0=none, 1=manager, 2=professional, 3=technician, 4=service worker, 5=agricultural, 6=industrial, 7=military; if housewife, retired or unemployed, we would like to know if you were employed in the past) ☐ 0-7

(☐ 0-7)

What I'd like to do is measure your blood pressure while you are sitting down, ask you a few questions about tobacco and alcohol, and then do your blood pressure again. Blood pressure (single measurement, seated): ☐☐☐ / ☐☐☐ SBP / DBP

Now, TOBACCO: Have you EVER smoked regularly (i.e. on most days for at least a year)? ☐ **Y=Yes, N=No**
If NO, then go directly to the ALCOHOL questions

AT ABOUT WHAT AGE DID YOU FIRST START SMOKING TOBACCO REGULARLY? ☐☐ years of age

Since then, have you ever STOPPED smoking for a year or more? ☐ Y=Yes, N=No

About how many cigarettes a day did you smoke last month? (0=none) ☐☐ daily

About how many cigars a day did you smoke last month? (0=none) ☐☐ daily

If NO SMOKING last month, at about what age did you last stop smoking? (blank = not applicable) ☐☐ years of age

Now I'd like to ask you some questions about ALCOHOL:

About how many days a week would you drink any rum or other spirits? (0=none or usually less than once a week) ☐ 0-7

On a day when you drink rum (or other spirits), about how many glasses ("lineas") would you drink? ☐☐ lineas

About how many days a week would you drink beer? (0=none or usually less than once a week) ☐ 0-7

On a day when you do drink beer, about how many bottles would you drink? ☐☐ bottles

Finally, MEDICAL MATTERS: I'd like to ask about various different illnesses. What I want to know for each illness is whether you've ever had medical attention for it. If you've never heard of the illness, then just say NO.

Asthma	☐ Y/N	Stroke (definite/probable)	☐ Y/N	Cirrhosis of the liver	☐ Y/N
Emphysema/C.O.P.D.	☐ Y/N	Myocardial infarction "	☐ Y/N	Chronic kidney disease	☐ Y/N
Diabetes	☐ Y/N	Angina pectoris "	☐ Y/N	Skin cancer	☐ Y/N
Peptic ulcer	☐ Y/N	Hypertension	☐ Y/N	Other cancer*	☐ Y/N

*If Other cancer, where in your body did the cancer start? ☐ 0-9
(1=mouth, 2=larynx, 3=lung, 4=oesphagus, 5=stomach, 6=pancreas, 7=cervix, 8=bladder, 9=breast, 0=other, more than 1 site, or not known)

Have you been taking any regular medication for more than 1 month? If Yes: What such medicines are you now taking? (Mark with an X)

Digoxin	☐	Nitrates	☐	Oral hypoglycaemic	☐	O.Contraceptive	☐	Ca. antagonist	☐
Beta blocker	☐	ACE inhibitor	☐	Insulin	☐	Antidepressant	☐	Bronchodilator	☐
Diuretic	☐	Aspirin	☐	Steroids	☐	Tranquillisers	☐	Antacid/H2	☐

THANK YOU VERY MUCH FOR YOUR ANSWERS. Now we just need one more blood pressure. Repeat blood pressure (single measurement, seated) ☐☐☐ / ☐☐☐ PAS / PAD

What was the last blood pressure in the patient's medical record **before** today?

Date of last BP (y,m,d) ☐☐☐☐☐☐ & ☐☐☐ / ☐☐☐ PAS / PAD

Please check that **ALL** the questions have been answered, so that there is no need to write back to you for extra details. **MANY THANKS!**

What is your approximate **HEIGHT** (either ask or measure)? ☐☐☐ cm

What is your approximate **WEIGHT** (either ask or measure)? ☐☐☐ kg

Date of interview ☐☐☐☐☐☐ (y,m.d)

Quality of co-operation (1=poor, 2=fair, 3=good) ☐ 1-3

Name of interviewer: please PRINT ____________________

Any comments: ....

Table S1. Prevalence of isolated systolic hypertension and isolated diastolic hypertension, by age and sex

			Hypertension (%)				
			Isolated systolic	Isolated diastolic	Both	Controlled	All
Age at entry (years)	No. of participants	SBP (mmHg): DBP(mmHg):	>=140 and <90	<140 and >=90	>=140 and >=90	<140 and <90	
**Male**							
35-39	9291		3.1	6.8	6.8	2.2	18.9
40-49	16 774		4.1	8.4	10.4	3.5	26.4
50-59	15 347		5.2	9.4	14.9	5.4	34.9
60-69	11 192		7.7	8.9	18.6	6.0	41.2
70-79	7560		9.6	8.7	18.0	6.2	42.5
**All**	**60 164**		**5.6**	**8.5**	**13.4**	**4.5**	**32.0**
**Female**							
35-39	12 301		2.0	4.5	4.8	2.8	14.1
40-49	21 651		3.4	6.6	9.5	5.2	24.7
50-59	19 014		5.8	8.9	17.1	8.8	40.6
60-69	13 920		8.7	8.7	21.9	10.1	49.4
70-79	9061		12.2	7.6	21.3	9.1	50.2
**All**	**75 947**		**5.8**	**7.4**	**14.4**	**7.1**	**34.7**
**Both**							
35-39	21 592		2.5	5.5	5.7	2.5	16.2
40-49	38 425		3.7	7.4	9.9	4.4	25.5
50-59	34 361		5.5	9.1	16.1	7.3	38.1
60-69	25 112		8.2	8.8	20.4	8.3	45.8
70-79	16 621		11.0	8.1	19.9	7.8	46.8
**Overall**	**136 111**		**5.7**	**7.9**	**13.9**	**6.0**	**33.5**

SBP = systolic blood pressure; DBP = diastolic blood pressure. Analyses are standardised for area, and where appropriate for age and sex. Exclusions as in Table 1.

Each percentage is that of the specified subgroup of the participants in each row.

Table S2. Prevalence of stage 1 hypertension and stage 2 hypertension, by age and sex

			Hypertension (%)			
			Stage 1	Stage 2	Controlled	All
Age at entry (years)	No. of participants	SBP (mmHg):<br>DBP(mmHg):	140-159<br>or 90-99	>=160<br>or >=100	<140<br>and <90	
**Male**						
35-39	9291		13.4	3.3	2.2	18.9
40-49	16 774		17.7	5.2	3.5	26.4
50-59	15 347		22.2	7.3	5.4	34.9
60-69	11 192		26.0	9.2	6.0	41.2
70-79	7560		26.7	9.6	6.2	42.5
**All**	**60 164**		**20.8**	**6.7**	**4.5**	**32.0**
**Female**						
35-39	12 301		9.0	2.3	2.8	14.1
40-49	21 651		14.8	4.7	5.2	24.7
50-59	19 014		22.9	8.9	8.8	40.6
60-69	13 920		27.5	11.7	10.1	49.4
70-79	9061		28.4	12.7	9.1	50.2
**All**	**75 947**		**19.9**	**7.7**	**7.1**	**34.7**
**Both**						
35-39	21 592		10.9	2.8	2.5	16.2
40-49	38 425		16.1	4.9	4.4	25.5
50-59	34 361		22.6	8.2	7.3	38.1
60-69	25 112		26.8	10.6	8.3	45.8
70-79	16 621		27.6	11.4	7.8	46.8
**Overall**	**136 111**		**20.3**	**7.2**	**6.0**	**33.5**

SBP = systolic blood pressure; DBP = diastolic blood pressure. Analyses are standardised for area, and where appropriate for age and sex.
Exclusions as in Table 1. Each percentage is that of the specified subgroup of the participants in each row.

Table S3. Proportion of participants with treated hypertension taking a single or multiple blood pressure-lowering medications, by age and sex

Age at entry (years)	Treated hypertensives, N	Treated hypertensives, N (%)			
		1 medication	2 medications	3 medications	4 medications
**Male**					
35-39	584	407 (69.7)	162 (27.7)	15 (2.6)	0 (0.0)
40-49	1728	1209 (70.0)	463 (26.8)	55 (3.2)	1 (0.1)
50-59	2502	1705 (68.1)	729 (29.1)	66 (2.6)	2 (0.1)
60-69	2240	1582 (70.6)	595 (26.6)	62 (2.8)	1 (0.0)
70-79	1490	1038 (69.7)	409 (27.5)	42 (2.8)	1 (0.1)
**Female**					
35-39	779	539 (69.2)	226 (29.0)	14 (1.8)	0 (0.0)
40-49	2938	2049 (69.7)	806 (27.4)	83 (2.8)	0 (0.0)
50-59	4712	3192 (67.7)	1381 (29.3)	139 (3.0)	0 (0.0)
60-69	4216	2939 (69.7)	1165 (27.6)	112 (2.7)	0 (0.0)
70-79	2568	1825 (71.1)	686 (26.7)	57 (2.2)	0 (0.0)
**Both**					
35-39	1363	946 (69.4)	388 (28.5)	29 (2.1)	0 (0.0)
40-49	4666	3258 (69.8)	1269 (27.2)	138 (3.0)	1 (0.0)
50-59	7214	4897 (67.9)	2110 (29.2)	205 (2.8)	2 (0.0)
60-69	6456	4521 (70.0)	1760 (27.3)	174 (2.7)	1 (0.0)
70-79	4058	2863 (70.6)	1095 (27.0)	99 (2.4)	1 (0.0)
**Overall**	**23 757**	**16 485 (69.4)**	**6622 (27.9)**	**645 (2.7)**	**5 (0.0)**

SBP = systolic blood pressure; DBP = diastolic blood pressure. Analyses are standardised for area, and where appropriate for age and sex.

Exclusions as in Table 1. Each percentage is that of the specified subgroup of the participants in each row.

Table S4. Proportion of participants with treated hypertension taking specific types of blood pressure-lowering medication, by age and sex

		% of treated hypertensives				% of treated hypertensives (single blood pressure-lowering medication only)				
Age at entry (years)	Treated hypertensives, N	ACE-inhibitors	Beta-blockers	Calcium channel blockers	Diuretics	ACE-inhibitors	Beta-blockers	Calcium channel blockers	Diuretics	Any
**Male**										
35-39	584	5.7	40.6	26.7	59.9	3.3	20.2	13.2	33.0	**69.7**
40-49	1728	5.7	40.0	27.7	59.9	2.9	19.5	13.8	33.8	**70.0**
50-59	2502	5.5	41.6	29.4	58.1	2.8	20.0	15.3	30.1	**68.1**
60-69	2240	5.0	34.7	33.8	58.7	2.3	16.0	18.8	33.5	**70.6**
70-79	1490	3.4	33.9	34.4	61.7	2.1	14.4	18.9	34.2	**69.7**
**Female**										
35-39	779	6.5	40.4	20.9	64.7	2.4	18.5	11.7	36.6	**69.2**
40-49	2938	4.7	41.6	23.8	63.0	2.2	19.5	11.7	36.3	**69.7**
50-59	4712	4.5	40.9	29.2	60.7	1.9	18.7	14.5	32.7	**67.7**
60-69	4216	4.6	35.7	32.7	60.0	2.1	16.2	17.8	33.6	**69.7**
70-79	2568	5.1	30.6	33.2	62.2	2.4	13.6	19.4	35.7	**71.1**
**Both**										
35-39	1363	6.2	40.5	23.4	62.7	2.8	19.2	12.3	35.1	**69.4**
40-49	4666	5.0	41.0	25.2	61.9	2.4	19.5	12.5	35.4	**69.8**
50-59	7214	4.9	41.1	29.2	59.8	2.2	19.1	14.8	31.8	**67.9**
60-69	6456	4.7	35.3	33.1	59.6	2.2	16.2	18.2	33.6	**70.0**
70-79	4058	4.5	31.8	33.6	62.0	2.3	13.9	19.2	35.1	**70.6**
**Overall**	**23 757**	**4.9**	**37.9**	**29.9**	**60.7**	**2.3**	**17.5**	**15.9**	**33.7**	**69.4**

SBP = systolic blood pressure; DBP = diastolic blood pressure. Analyses are standardised for area, and where appropriate for age and sex.

Exclusions as in Table 1. Each percentage is that of the specified subgroup of the participants in each row.

Table S5. Baseline characteristics of the 23 114 study participants that were resurveyed*

	Men (10 183)	Women (12 931)	All (23 114)
Age at entry (years), n (%)			
35-39	1784 (18)	2352 (18)	4136 (18)
40-49	2717 (27)	3708 (29)	6425 (28)
50-59	2813 (28)	3362 (26)	6175 (27)
60-69	1788 (18)	2198 (17)	3986 (17)
70-79	1081 (11)	1311 (10)	2392 (10)
Age (years), mean (SD)	53 (12)	53 (12)	53 (12)
Blood pressure (mm Hg), mean (SD)			
Systolic	126 (17)	124 (20)	125 (18)
Diastolic	81 (10)	79 (12)	80 (11)
Area, n (%)†			
Matanzas	10 183 (100)	12 931 (100)	23 114 (100)
Camagüey	0 (0)	0 (0)	0 (0)
Pinar del Río	0 (0)	0 (0)	0 (0)
Ciudad de La Habana	0 (0)	0 (0)	0 (0)
La Habana	0 (0)	0 (0)	0 (0)
Educational level, n (%)			
Less than primary	710 (7)	1125 (9)	1835 (8)
Primary	2700 (27)	4207 (33)	6907 (30)
Lower secondary	3002 (29)	3694 (29)	6696 (29)
High school/technical college	2862 (28)	2950 (23)	5812 (25)
University	909 (9)	955 (7)	1864 (8)
Ethnicity			
White	7919 (78)	9947 (77)	17 866 (77)
Black	1606 (16)	1945 (15)	3551 (15)
Mixed	649 (6)	1024 (8)	1673 (7)
Other	9 (<1)	15 (<1)	24 (<1)
Prior cardiovascular disease			
No	9706 (95)	12 404 (96)	22 110 (96)
Yes	477 (5)	527 (4)	1004 (4)
Season of baseline survey, n (%)†			
Spring	5135 (50)	7038 (54)	12 173 (53)
Summer	3376 (33)	3521 (27)	6897 (30)
Autumn	599 (6)	837 (6)	1436 (6)
Winter	1073 (11)	1535 (12)	2608 (11)
Ever smoked tobacco, n (%)	5761 (57)	3937 (30)	9698 (42)
At least weekly alcohol consumption, n (%)	2215 (22)	211 (2)	2426 (10)
BMI (kg/m^2), mean (SD)	24.6 (3.8)	25.2 (4.5)	24.9 (4.2)

*Exclusion as in Table 1, with further exclusion of participants with missing or implausible blood pressure values at resurvey.
†Spring indicates March to May; Summer, June to August; Autumn, September to November; Winter, December to February.

Table S6. Prevalence, diagnosis, treatment and control of hypertension at baseline among resurveyed participants, by age and sex

Age at baseline (years)	No. of participants	Prevalence of hypertension, %	Diagnosed among hypertensives, %	Treated among diagnosed, %	Controlled	
					Among treated, %	Among all hypertensives, %
**Male**						
35-39	1784	18.8	37.5	55.6	42.9	8.9
40-49	2717	28.3	41.3	61.9	38.6	9.9
50-59	2813	37.5	49.3	68.5	32.9	11.1
60-69	1788	42.7	49.7	73.2	28.4	10.3
70-79	182	45.6	54.2	62.2	35.7	12.0
**All**	**9 284**	**32.2**	**46.0**	**66.7**	**33.7**	**10.4**
**Female**						
35-39	2352	12.8	48.5	66.4	45.4	14.6
40-49	3708	26.2	62.6	73.1	37.8	17.3
50-59	3362	43.6	68.0	75.5	34.0	17.5
60-69	2198	51.8	70.9	74.4	27.6	14.6
70-79	234	53.0	69.4	80.2	27.5	15.3
**All**	**11 854**	**34.0**	**66.2**	**74.3**	**33.2**	**16.3**
**Both**						
35-39	4136	15.5	42.6	61.3	44.3	11.6
40-49	6425	27.1	52.8	69.1	38.1	13.9
50-59	6175	40.9	60.5	73.2	33.7	14.9
60-69	3986	47.8	62.6	74.0	27.9	12.9
70-79	416	49.7	63.3	74.0	29.9	14.0
**All (40-79)**	**17 002**	**37.5**	**59.0**	**72.5**	**32.7**	**14.0**
**All (35-79)**	**21 138**	**33.2**	**57.6**	**71.7**	**33.4**	**13.8**

SBP = systolic blood pressure; DBP = diastolic blood pressure. Analyses are standardised for area, and where appropriate for age and sex. Exclusions as in Table 1, with further exclusion of participants with missing or implausible blood pressure values at resurvey, and those aged less than 40 or 80 and over at resurvey.

Each percentage is that of the specified subgroup of the participants in each row.

Table S7. Prevalence, diagnosis, treatment and control of hypertension at among resurveyed participants, by age and sex

Age at resurvey (years)	No. of participants	Prevalence of hypertension, %	Diagnosed among hypertensives, %	Treated among diagnosed, %	Controlled	
					Among treated, %	Among all hypertensives, %
**Male**						
40-49	2147	29.5	53.6	79.6	67.4	28.8
50-59	2811	38.1	55.9	78.0	56.3	24.5
60-69	2687	42.9	62.3	76.5	49.3	23.5
70-79	1639	45.0	64.7	73.0	50.9	24.0
**All**	**9284**	**37.4**	**57.8**	**77.5**	**57.6**	**25.6**
**Female**						
40-49	2842	25.3	59.4	83.2	72.5	35.8
50-59	3733	42.2	65.5	77.7	59.4	30.2
60-69	3183	51.7	75.6	73.2	51.9	28.8
70-79	2096	56.3	76.8	69.4	42.5	22.6
**All**	**11 854**	**40.5**	**67.2**	**77.4**	**59.8**	**30.7**
**Both**						
40-49	4989	27.2	56.6	81.6	70.2	32.5
50-59	6544	40.4	61.5	77.8	58.3	27.9
60-69	5870	47.8	70.4	74.4	51.0	26.7
70-79	3735	51.3	72.1	70.6	45.5	23.2
**Overall**	**21 138**	**39.1**	**63.1**	**77.4**	**58.8**	**28.5**

SBP = systolic blood pressure; DBP = diastolic blood pressure. Analyses are standardised for area, and where appropriate for age and sex. Exclusions as in Table 1, with further exclusion of participants with missing or implausible blood pressure values at resurvey, and those aged less than 40 or 80 and over at resurvey.
Each percentage is that of the specified subgroup of the participants in each row.

Table S8. Proportion of participants with treated hypertension at resurvey taking a single or multiple blood pressure-lowering medications, by age and sex

Age at resurvey (years)	Treated with hypertensives, N	Treated hypertensives, N (%)			
		1 medication	2 medications	3 medications	4 medications
**Male**					
40-49	280	173 (61.8)	98 (35.0)	9 (3.2)	0 (0.0)
50-59	495	312 (63.0)	161 (32.5)	22 (4.4)	0 (0.0)
60-69	583	347 (59.5)	204 (35.0)	31 (5.3)	1 (0.2)
70-79	368	231 (62.8)	127 (34.5)	10 (2.7)	0 (0.0)
**Female**					
40-49	377	228 (60.5)	133 (35.3)	15 (4.0)	1 (0.3)
50-59	863	560 (64.9)	276 (32.0)	26 (3.0)	1 (0.1)
60-69	938	617 (65.8)	295 (31.5)	25 (2.7)	1 (0.1)
70-79	653	434 (66.5)	198 (30.3)	20 (3.1)	1 (0.2)
**Both**					
40-49	657	401 (61.0)	231 (35.2)	24 (3.7)	1 (0.2)
50-59	1358	872 (64.2)	437 (32.2)	48 (3.5)	1 (0.1)
60-69	1521	964 (63.4)	499 (32.8)	56 (3.7)	2 (0.1)
70-79	1021	665 (65.1)	325 (31.8)	30 (2.9)	1 (0.1)
**Overall**	**4557**	**2902 (63.7)**	**1492 (32.7)**	**158 (3.5)**	**5 (0.1)**

SBP = systolic blood pressure; DBP = diastolic blood pressure. Analyses are standardised for area, and where appropriate for age and sex.

Exclusions as in Table 1. Each percentage is that of the specified subgroup of the participants in each row.

Table S9. Baseline characteristics of the 127 253 study participants in the prospective analyses*

	Men (56 335)		Women (70 918)		All (127 253)	
Age at entry (years), n (%)						
35-39	9156	(16)	12 144	(17)	21 300	(17)
40-49	16 316	(29)	21 002	(30)	37 318	(29)
50-59	14 442	(26)	17 656	(25)	32 098	(25)
60-69	10 016	(18)	12 392	(17)	22 408	(18)
70-79	6405	(11)	7724	(11)	14 129	(11)
Age (years), mean (SD)	53	(12)	53	(12)	53	(12)
Blood pressure (mm Hg), mean (SD)						
Systolic	125	(14)	124	(16)	124	(15)
Diastolic	81	(9)	79	(10)	80	(10)
Area, n (%)						
Matanzas	25 067	(44)	30 719	(43)	55 786	(44)
Camagüey	22 705	(40)	28 624	(40)	51 329	(40)
Pinar del Río	4522	(8)	5441	(8)	9963	(8)
Ciudad de La Habana	2842	(5)	4578	(6)	7420	(6)
La Habana	1199	(2)	1556	(2)	2755	(2)
Educational level, n (%)						
Less than primary	2919	(5)	4899	(7)	7818	(6)
Primary	13 365	(24)	20 943	(30)	34 308	(27)
Lower secondary	15 243	(27)	19 932	(28)	35 175	(28)
High school/technical college	19 100	(34)	18 815	(27)	37 915	(30)
University	5708	(10)	6329	(9)	12 037	(9)
Ethnicity						
White	43 559	(77)	54 403	(77)	97 962	(77)
Black	8243	(15)	10 098	(14)	18 341	(14)
Mixed	4341	(8)	6175	(9)	10 516	(8)
Other	192	(<1)	242	(<1)	434	(<1)
Prior cardiovascular disease						
No	56 335	(100)	70 918	(100)	127 253	(100)
Yes	0	(0)	0	(0)	0	(0)
Season of baseline survey, n (%)†						
Spring	11 778	(21)	15 123	(21)	26 901	(21)
Summer	18 425	(33)	22 280	(31)	40 705	(32)
Autumn	11 380	(20)	14 991	(21)	26 371	(21)
Winter	14 752	(26)	18 524	(26)	33 276	(26)
Ever smoked tobacco, n (%)	33 302	(59)	23 364	(33)	56 666	(45)
At least weekly alcohol consumption, n (%)	16 553	(29)	3266	(5)	19 819	(16)
BMI (kg/m^2), mean (SD)	24.1	(3.7)	24.4	(4.4)	24.3	(4.1)

*Excludes participants with missing information, those with implausible blood pressure values, or those without follow-up at age 35-79 year. In addition excludes those with prior history at baseline of angina, myocardial infarction or stroke.

†Spring indicates March to May; Summer, June to August; Autumn, September to November; Winter, December to February.

Figure S1. Age− and sex−specific relevance of hypertension to cardiovascular mortality

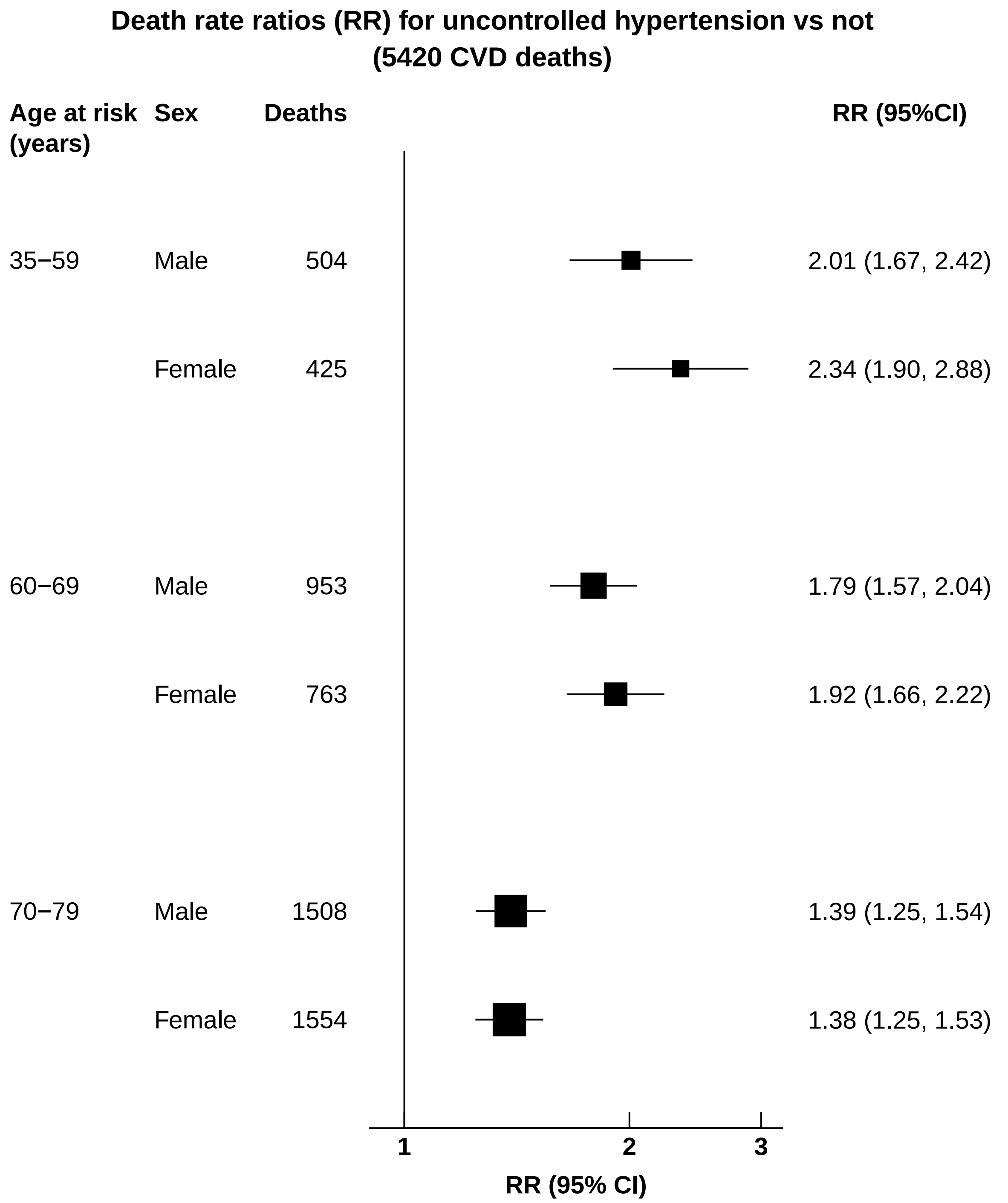

Death rate ratios (RRs) are calculated for participants with vs without uncontrolled hypertension.
RRs are adjusted for area, level of education, smoking, alcohol, body mass index and age (in 5−year bands) within each age group.
Exclusions as in Table 1 with further exclusion of those participants with a prior cardiovascular disease at baseline.
For each RR, the area of the square is inversely proportional to the variance of the log risk.

Table S10. Prevalence and control of hypertension at baseline using four different definitions of hypertension*

Age at entry (years)	No. of participants	Standard definition		Alternative definition 1		Alternative definition 2		Alternative definition 3	
		Prevalence, %	Control, %	Prevalence, %	Control, %	Prevalence, %	Control, %	Prevalence, %	Control, %
**Male**									
35-39	9291	18.9	11.7	19.4	13.9	21.8	23.3	22.3	24.9
40-49	16 774	26.4	13.1	27.0	15.4	29.3	21.9	30.0	23.8
50-59	15 347	34.9	15.4	36.0	17.9	38.1	22.4	39.1	24.6
60-69	11 192	41.2	14.5	42.9	18.0	44.7	21.2	46.5	24.2
70-79	7560	42.5	14.5	45.2	19.6	45.9	20.9	48.6	25.3
**All**	**60 164**	**32.0**	**14.2**	**33.2**	**17.3**	**35.2**	**21.9**	**36.4**	**24.5**
**Female**									
35-39	12 301	14.1	19.6	14.8	23.4	17.6	36.0	18.3	38.4
40-49	21 651	24.7	21.0	25.8	24.1	29.2	33.0	30.2	35.3
50-59	19 014	40.6	21.8	42.1	24.5	46.2	31.1	47.6	33.3
60-69	13 920	49.4	20.5	50.9	23.0	55.0	28.7	56.6	30.7
70-79	9061	50.2	18.1	52.6	21.8	55.4	25.7	57.8	28.9
**All**	**75 947**	**34.7**	**20.6**	**36.1**	**23.5**	**39.6**	**30.4**	**41.0**	**32.7**
**Both**									
35-39	21 592	16.2	15.5	16.8	18.6	19.5	29.7	20.1	31.8
40-49	38 425	25.5	17.4	26.3	20.1	29.2	28.1	30.1	30.2
50-59	34 361	38.1	19.2	39.4	21.9	42.6	27.7	43.9	29.9
60-69	25 112	45.8	18.1	47.4	21.0	50.5	25.8	52.1	28.1
70-79	16 621	46.8	16.6	49.3	20.9	51.2	23.8	53.7	27.4
**Overall**	**136 111**	**33.5**	**17.9**	**34.8**	**20.9**	**37.6**	**26.9**	**38.9**	**29.3**

SBP = systolic blood pressure; DBP = diastolic blood pressure. Analyses are standardised for area, and where appropriate for age and sex. Exclusions as in Table 1.

Each percentage is that of the specified subgroup of the participants in each row.

* Standard definition: Measured SBP >= 140 mm Hg or DBP >= 90 mm Hg or (diagnosed AND treated with any of ace inhibitors, beta-blockers, ca antagonist, diuretics).

Alternative definition 1: Measured SBP >= 140 mm Hg or DBP >= 90 mm Hg or treated with any of ace inhibitors, beta-blockers, ca antagonist, diuretics.

Alternative definition 2: Measured SBP >= 140 mm Hg or DBP >= 90 mm Hg or diagnosed.

Alternative definition 3: Measured SBP >= 140 mm Hg or DBP >= 90 mm Hg or (diagnosed AND/OR treated with any of ace inhibitors, beta-blockers, ca antagonist, diuretics).
